# Biomechanical effects of length and angle of the subcutaneous tunnel on epidural catheter stability in a porcine model

**DOI:** 10.1038/s41598-026-71055-7

**Published:** 2026-09-10

**Authors:** Jan Pennig, Sarah N. Schmidt, Axel Schmutz, Johannes Hell

**Affiliations:** 1 Department of Anesthesiology and Critical Care, Medical Center, University of Freiburg, Hugstetter Str. 55, 79106 Freiburg, Germany; 2 Member of Faculty of Medicine, University of Freiburg, Freiburg, Germany

**Keywords:** Epidural catheter, Epidural catheter fixation, Epidural catheter dislocation, Catheter stabilization, Diseases, Engineering, Health care, Medical research

## Abstract

Epidural catheter dislocation can lead to severe complications, especially in anticoagulated patients. Fixation techniques vary, and the role of subcutaneous tunneling and the biomechanical effects of length or angle towards dislocation forces of the tunnel remain unknown. This in vitro study evaluated the contribution of length and angle of subcutaneous tunneling (2–5 cm at six various angles relative to dislocation force) compared to different adhesive fixation methods (transparent film dressing, adhesive strips 10–20 cm) on epidural catheter stability. Dislocation forces were measured in a porcine skin model using a motorized traction system and a force transducer. The mean dislodgement force for tunneled catheters was 0.86 ± 0.3 N. This was significantly lower than the forces measured using transparent film dressing (9.89 ± 2.65 N; *p* < 0.001) or adhesive strips (10 cm: 8.03 ± 2.3 N; 20 cm: 10.92 ± 0.84 N; all *p* < 0.001). Tunnel length and angle had no significant impact on catheter stability whereas increasing the length of the adhesive strip significantly increased the dislodgement force (*p* < 0.001). In conclusion, subcutaneous tunneling of epidural catheters provides limited mechanical benefit on catheter stability under standardized conditions in a porcine model whereas adhesive fixation generates substantially greater resistance to immediate catheter dislocation.

**Supplementary Information:**

The online version contains supplementary material available at https://doi.org/10.1038/s41598-026-71055-7.

## Introduction

**Fig. 1:**
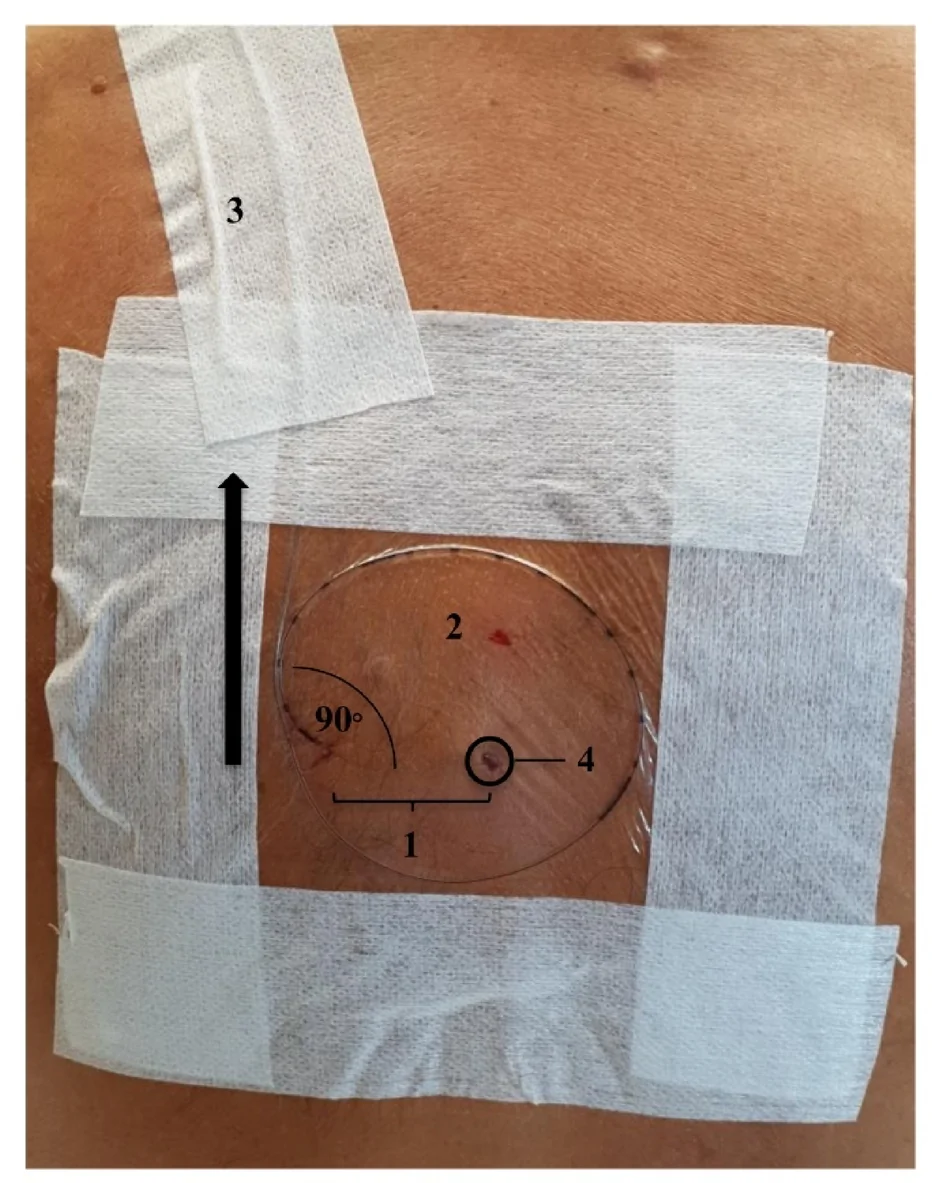
Representative picture of our clinical standard fixation for epidural catheters, that consists of 3 components: a subcutaneous tunnel (1), a transparent film dressing (2) with an adhesive strip framework over the looped catheter, and an adhesive strip (3) applied alongside the catheter´s path towards the shoulder. The catheter is tunneled at a 90° angle towards the estimated direction of dislocation force (black arrow). Insertion site (4).

Epidural anesthesia provides excellent analgesia in major surgery and during labor. It may reduce mortality in abdominal and thoracic surgery and may even affect tumor progression in oncological surgery^1^. However, epidural catheters are associated with an inherent risk of epidural bleeding and hematoma, particularly during placement and removal of the catheter. Accidental dislodgement may increase the risk of epidural hematoma, particularly in patients under anticoagulant medication, due to inadequate pausing^2^ and may expose patients to risks associated with replacing the epidural catheter if necessary. Therefore, proper securing of the catheter is crucial to prevent accidental migration or dislodgement. Nevertheless, previous studies report accidental epidural catheter migration rates of up to 56% with a wide variety of fixation techniques^3–8^. At our institution, we use a combination of subcutaneous tunneling and a transparent film dressing over the insertion site, combined with adhesive strips along the catheter´s path (Fig. 1).

However, the data on whether supplementary subcutaneous tunneling reliably affects catheter migration is conflicting but at the same time tunneling is an additional unpleasant experience for patients^9^. While some studies suggest that tunneling may reduce the risk of catheter dislocation^5,7^, others have mixed results^4,8^ or fail to demonstrate a significant benefit^10^. This discrepancy underscores the need for further investigation of the mechanics of catheter fixation.

Our goal was to characterize the individual biomechanical effects of our three fixation components to overall catheter stability, focusing particularly on the length and direction of the subcutaneous tunnel. We hypothesized that the required dislocation forces would increase with the length and angle of the subcutaneous tunnel relative to the applied force.

## Methods

This prospective non-blinded experimental bench study was conducted by the Department of Anesthesiology and Critical Care, Medical Center, University of Freiburg, Freiburg, Germany from May 2024 to November 2024. According to the local Animal Welfare Committee (Regierungspraesidium Freiburg) approval was not required, and no concerns were raised regarding animal welfare laws. An ethical approval and patient consent statement was not needed for this study.

### Experimental setup

We measured the peak force required to dislocate an epidural catheter. According to the three main components of our standard clinical fixation (Fig. 1)—the subcutaneous tunnel, the transparent film dressing over the insertion site, and the adhesive strip on the catheter—we measured the contributions of each component to overall fixation. For the subcutaneous tunnel, we investigated how the tunnel’s length and direction impact the required dislocation force at six different angles. Additionally, we evaluated the impact of the adhesive strip length on dislocation force. Therefore, we purchased porcine skin flaps with subcutaneous fat from a local butcher of freshly slaughtered animals. The flaps were warmed to 37 °C using a forced-air warming blanket (Cocoon CWS 5000, Care Essentials, North Geelong, Australia). The flaps were fixed to the ground with pins to prevent longitudinal movement when traction was applied to the catheter.

### Force measurement

**Fig. 2:**
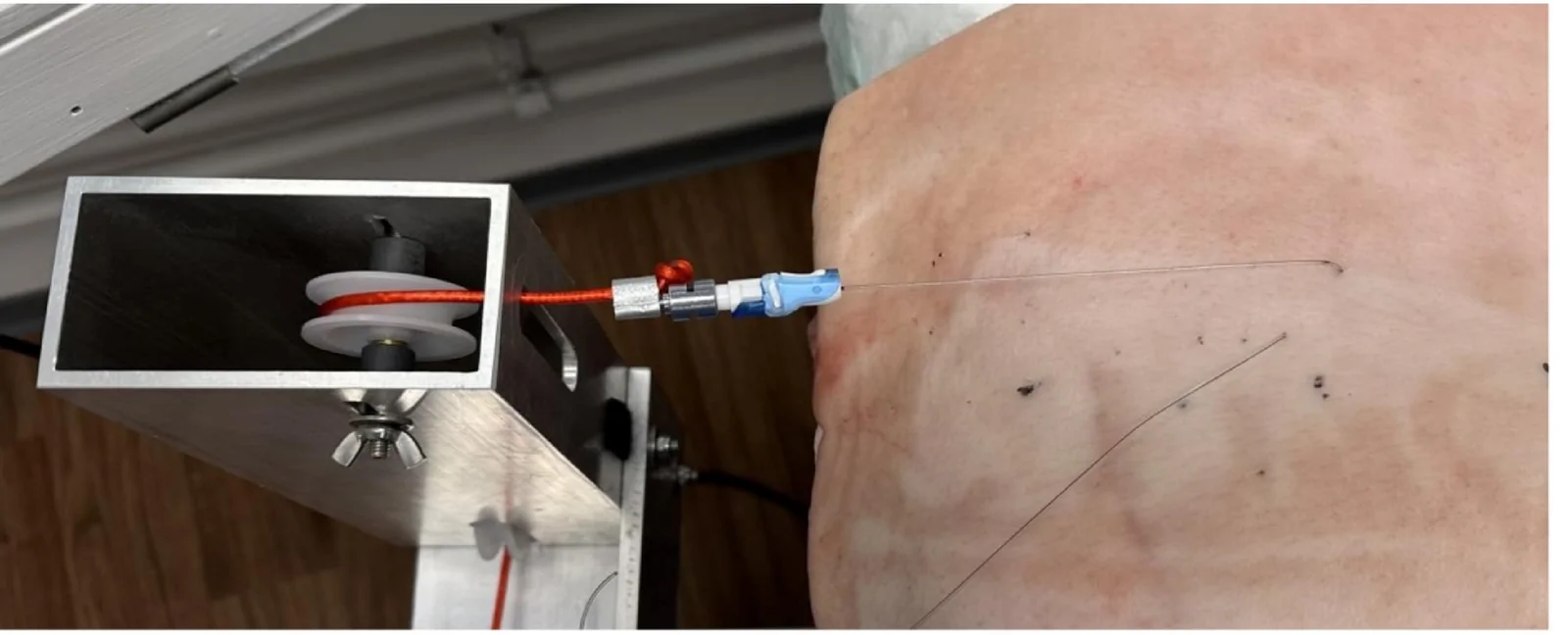
Experimental setup. Epidural catheter connected to a red rope via luer lock clamping adapter. The rope passes two deflection rollers and is then connected to a force transducer.

The luer lock plug of the epidural catheter clamping adapter was connected to a rope via a luer lock adapter. The rope passed through two deflection rollers and connected to a force transducer (PSD-S1 Load cell, Zhengzhou Pushton Electronic Instruments Co., Ltd., Zhengzhou, China) with a maximum load capacity up to 50 kg and an accuracy class C2 with an expected maximum measurement error of ± 0.15 N. The force transducer was connected to a drawbar spindle. The force transducer has been validated for retest reliability in previous experiments conducted by our department^11^. To increase tension on the catheter, a direct current motor turned the drawbar spindle at a constant rotational speed (Fig. 2). Force was continuously recorded using custom software in LabVIEW 7.1 (National Instruments, Austin TX). The raw force data were analyzed using MATLAB (MathWorks^®^, Natick, MA, USA). After filtering the continuous force recordings, we determined the peak force before epidural catheter dislocation. The maximum dislocation force of each fixation method was measured eight times. A complete disassembly and reassembly of the laboratory setup was performed between repetitions.

### Tunneling of epidural catheters

**Fig. 3:**
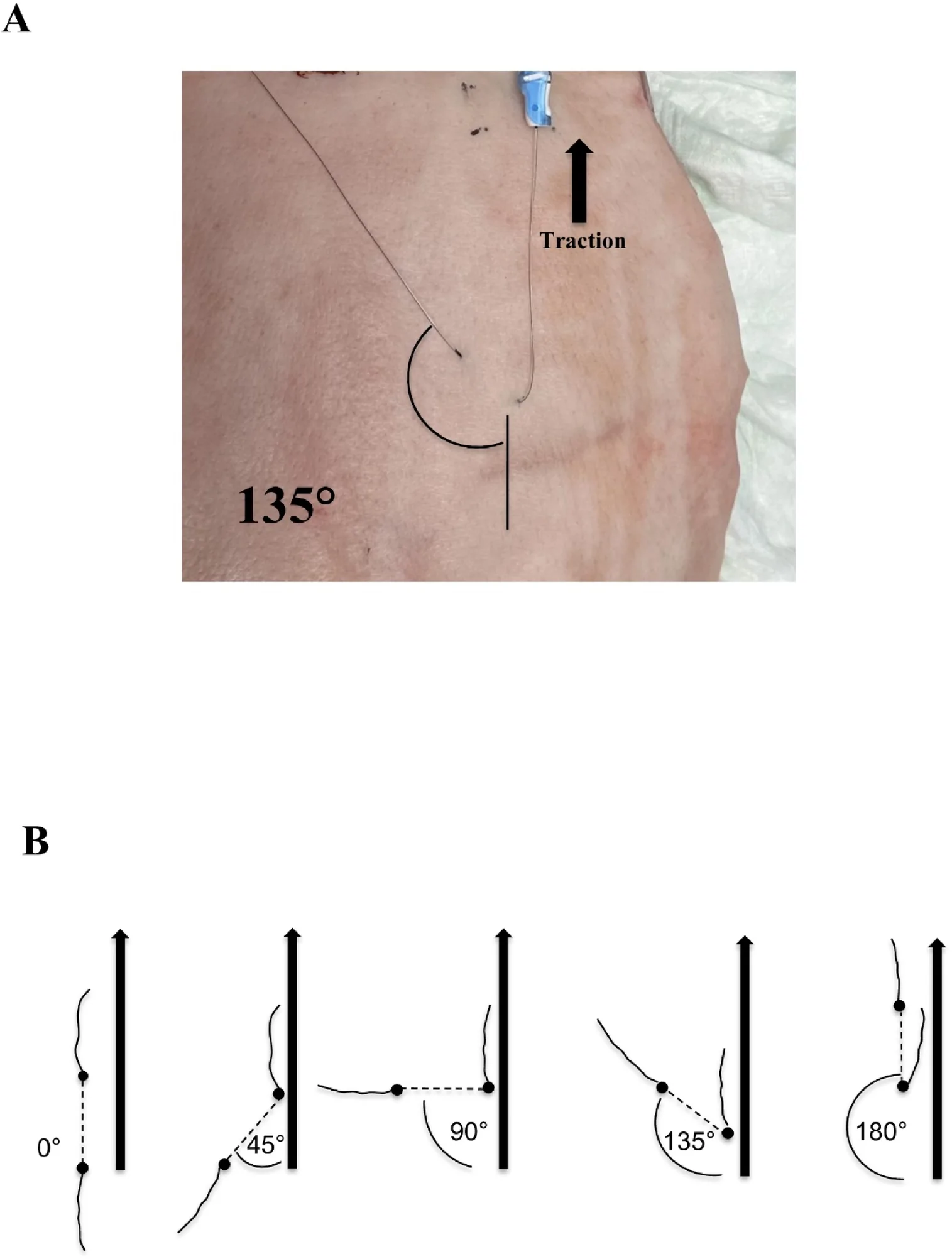
**(a)** Representative picture of a 2 cm subcutaneously tunneled epidural catheter with traction applied at a 135° angle. **(b)** Schematic illustration demonstrating the application of traction on epidural catheters at various angles relative to the subcutaneous tunnel. Dashed lines indicate the subcutaneous tunnel, black dots mark entrance and exit of the tunnel, solid lines represent the epidural catheter, and black arrows show the direction of applied traction.

Subcutaneous tunneling was performed using a 17-gauge i.v. cannula (Vasofix^®^ Safety, B Braun, Melsungen, Germany), as is done in our clinical practice. 20-gauge epidural catheters were taken from Pajunk^®^ Epidural sets (REF 001151 − 616, Pajunk^®^ GmbH, Geisingen, Germany). The catheters were connected to the corresponding clamping adapter with a luer lock system for connection to the force transducer. Traction was applied horizontally and parallel to the skin surface. The tunnel length was either 2–5 cm. Each length was divided into five subgroups, where traction on the catheter was applied directly in line with the course of the tunnel (0°) or with an angle of 45°, 90°, 135° or 180° (Fig. 3a,b).

### Fixation with adhesive strips and with transparent film dressing

**Fig. 4:**
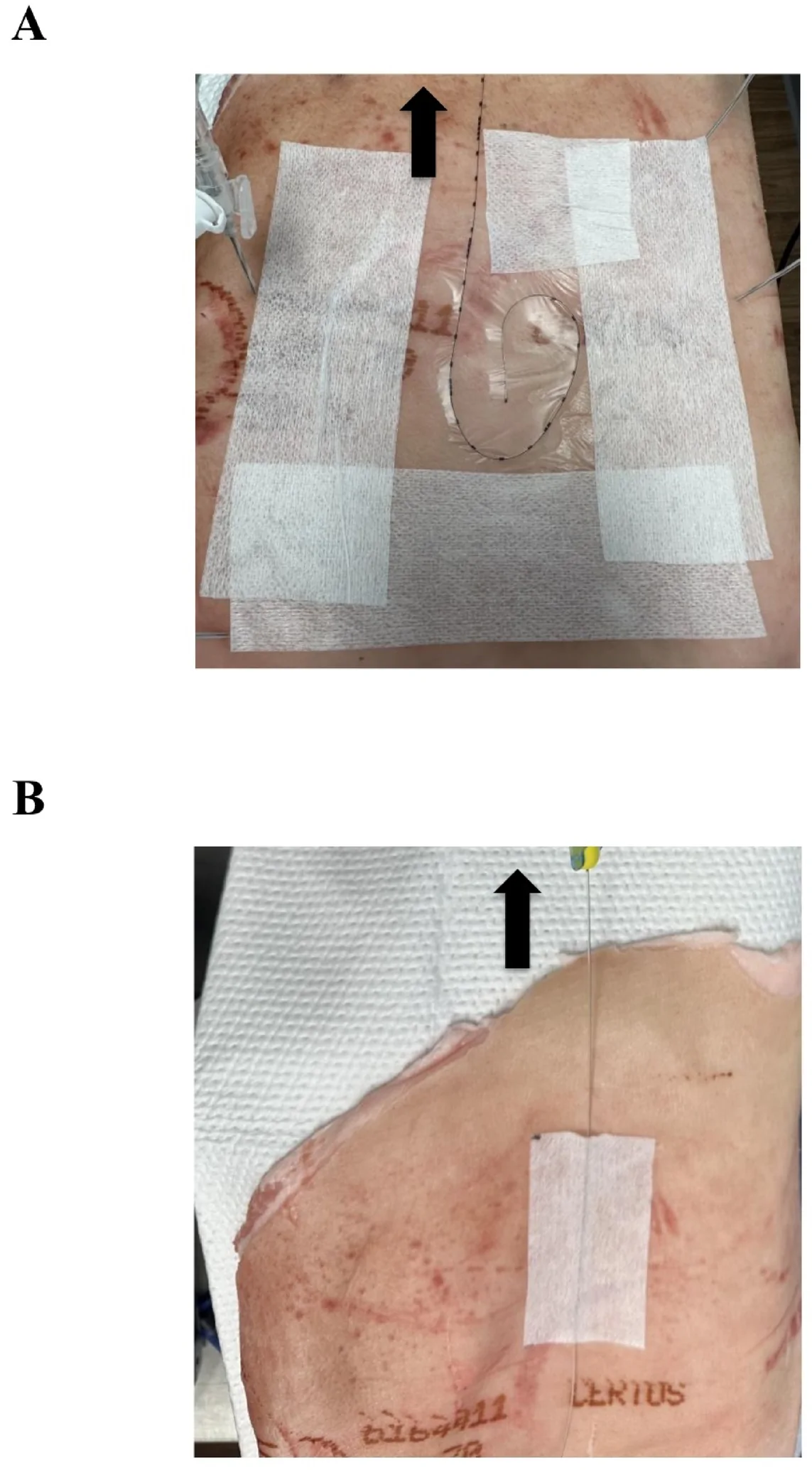
Representative examples of epidural catheters secured with **(a)** a transparent film dressing with a Fixomull^®^ adhesive strip framework or **(b)** a 10-cm-long Fixomull^®^ adhesive strip. Black arrows indicate the direction of traction.

To measure the dislocation force required to remove a catheter secured by adhesive strips, the catheter was attached to porcine skin with either 10–20 cm of Fixomull^®^ adhesive strips (BSN Medical GmbH, Hamburg, Germany). The adhesive strips were positioned along the catheter (Fig. 4a). To evaluate the contribution of transparent film dressing (Tegaderm™ Film, Solventum Germany GmbH, Kamen, Germany) to the catheter’s overall stability, a looped catheter was fixed with a transparent film dressing on the skin of our model. The edge of the transparent film dressing was fastened with a Fixomull^®^ framework, as is done in clinical practice. At the point where the catheter exited the transparent film dressing, the Fixomull^®^ framework was interrupted to assess the friction effect of the transparent film dressing alone (Fig. 4b). Traction was applied along the catheter’s path. All adhesive materials were newly opened for each experiment and always attached by the same investigator.

### Sample size

We considered an effect of the angle of 10% on the dislocation force to be clinically relevant. Therefore, using an ANOVA in our 5 × 2 design (angles x lengths) to achieve a power of 0.8 and an alpha level of 0.05 for the effect of the angle with an estimated effect size of 0.4, 80 samples were needed. Divided into ten groups, 8 measurements per angle and length were necessary.

### Endpoints of the study

The primary endpoint was the effect of tunnel length and angle of the tunnel on the dislocation force. Secondary endpoints included the effect of adhesive strips and of transparent film dressing on the dislocation forces.

### Statistical analysis

Descriptive variables are given as mean ± SD, comparative data are presented as mean difference (MD) and 95% confidence interval [CI95%: 5% to 95%] as appropriate. In BoxPlots, the box shows the first and the third quartile. The horizontal line within the box represents the median. The whiskers below and above the box show the minimum and maximum.

Levene´s test was used to test for equality of variances. When equality of variances was given, One-way ANOVA followed by Bonferroni´s post-hoc test was used to compare between groups. When Levene´s test was significant or when group sizes were differing, Welch-ANOVA was used. To test more than one independent variable, a multivariate ANOVA was used. The linear regression model was used to analyze the relationship between two variables. Two groups were compared with Student´s t-test. Results with *p* < 0.05 were considered statistically significant. Data was analyzed using SPSS (version 29.0.0, IBM, Armonk, NY, USA). G*Power was used for sample size calculation (G*Power 3.1.9.2. HHU Düsseldorf).

## Results

**Fig. 5:**
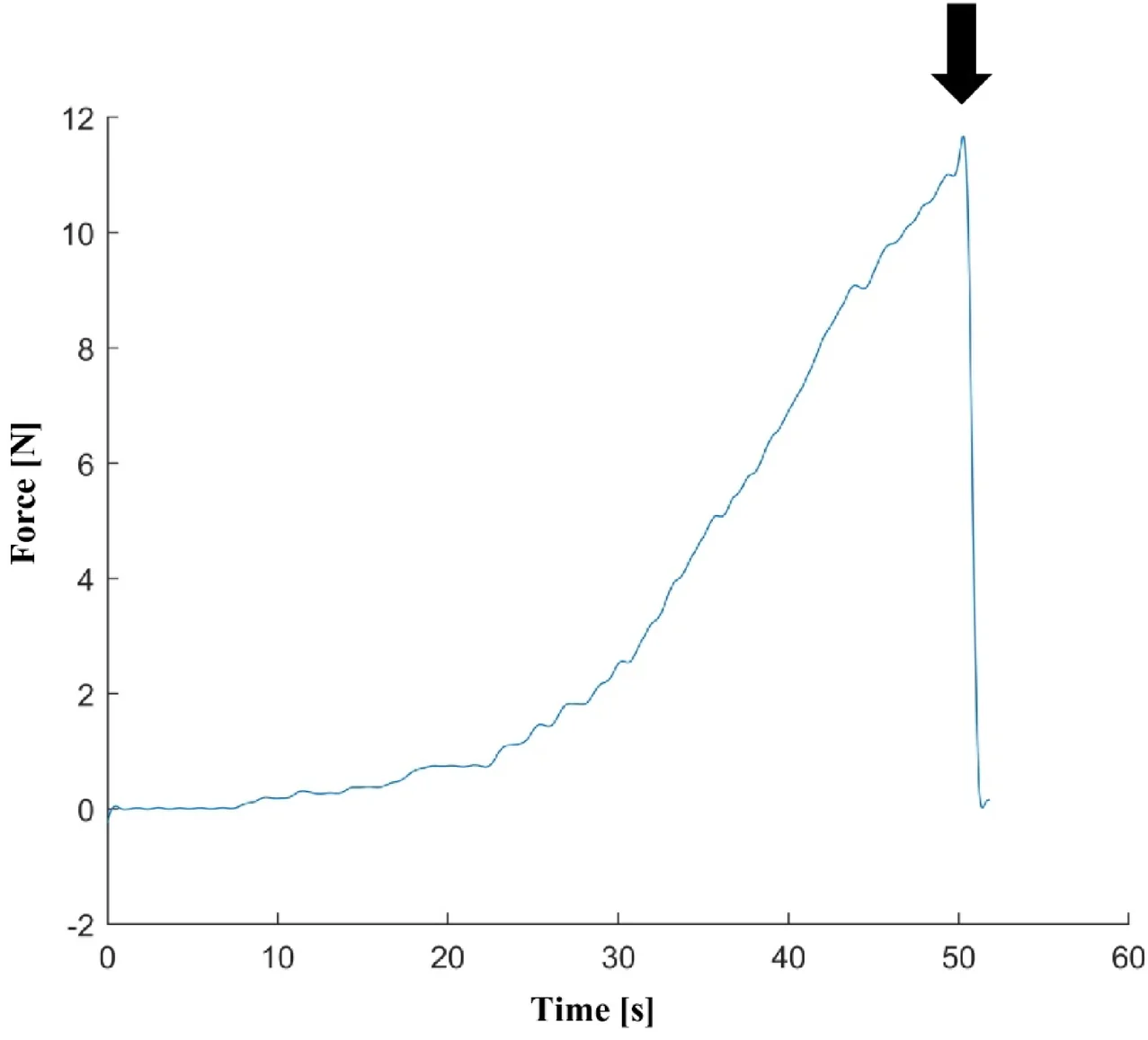
Representative curve derived from continuous force measurement recorded from the start of traction until catheter dislocation. Arrow indicates time point of dislocation.

Continuously augmented traction resulted in an increase in force until a peak was reached followed by dislocation and subsequent rapid decline (Fig. 5).

### Epidural catheter tunneling: The effects of angle and tunnel length on required dislocation forces

Although the overall multivariate ANOVA indicated a statistically significant effect of tunnel angle (F = 2.654, *p* = 0.04), post hoc analyses with Bonferroni corrected p-values revealed no significant difference between different angles (Supp_Tab 1). Furthermore, testing did not reveal significant differences between the dislocation forces regarding tunnel length (F = 3.251, *p* = 0.08) and no interaction effects between tunnel length and angle were found (F = 2.258, *p* = 0.07).

**Fig. 6:**
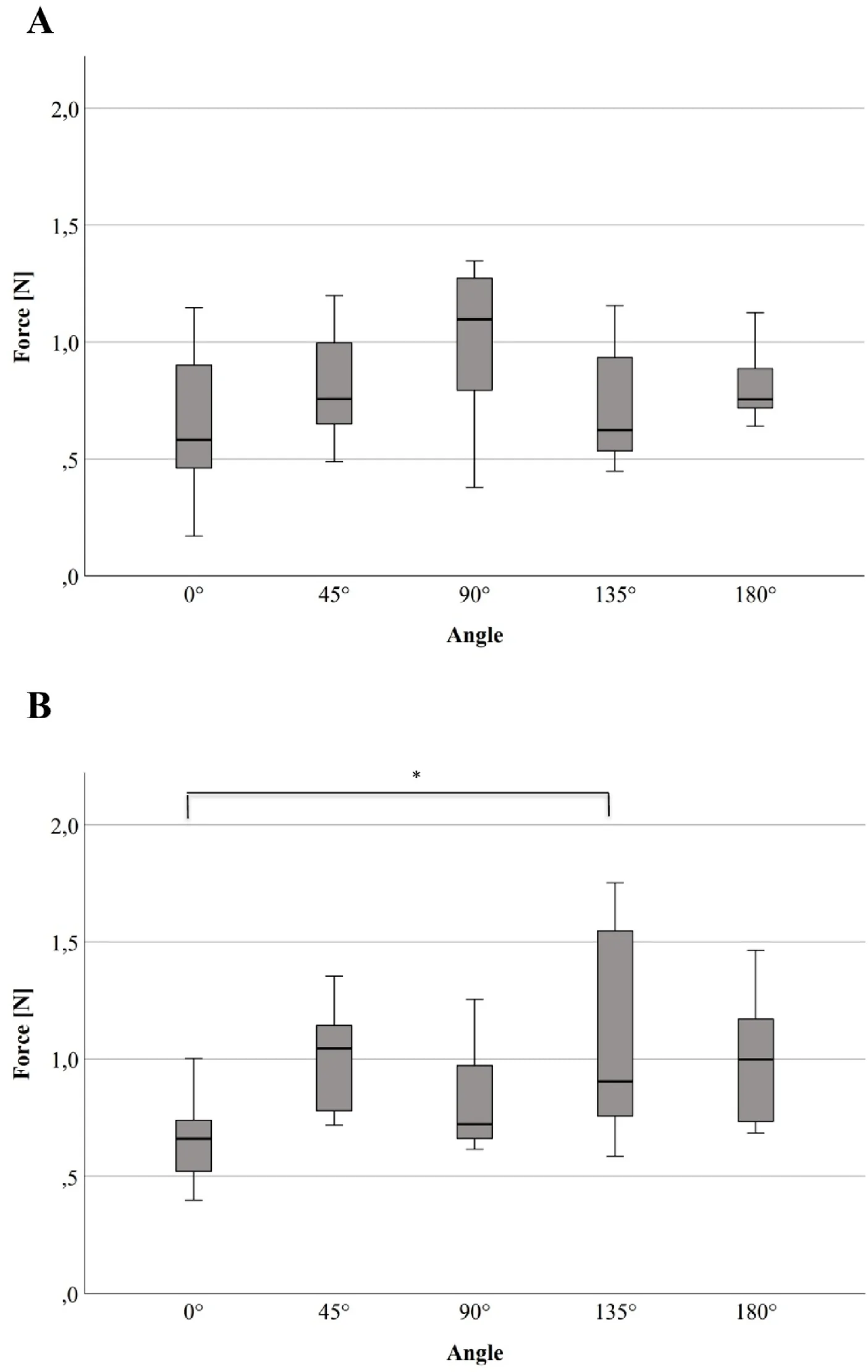
BoxPlot showing required dislocation forces at **(a)** 2 cm or **(b)** 5 cm tunnel length with traction applied at an angle of 0° (*n* = 16), 45° (*n* = 16), 90° (*n* = 16), 135° (*n* = 16) and 180° (*n* = 16). * = *p* < 0.05.

At a tunnel length of 2 cm the force required for epidural catheter dislocation ranged from 0.65 ± 0.33 N to 1.00 ± 0.33 N with no significant difference observed based on the angle of traction applied (Fig. 6a). At a tunnel length of 5 cm likewise, the required dislocation forces varied between 0.66 ± 0.20 N and 1.10 ± 0.22 N. These forces did not differ significantly from each other, except for a slightly significant difference between an angle of 135° and 0° (MD 0.439 [CI95%: 0.01 to 0.87]; *p* = 0.049 Fig. 6b).

**Fig. 7:**
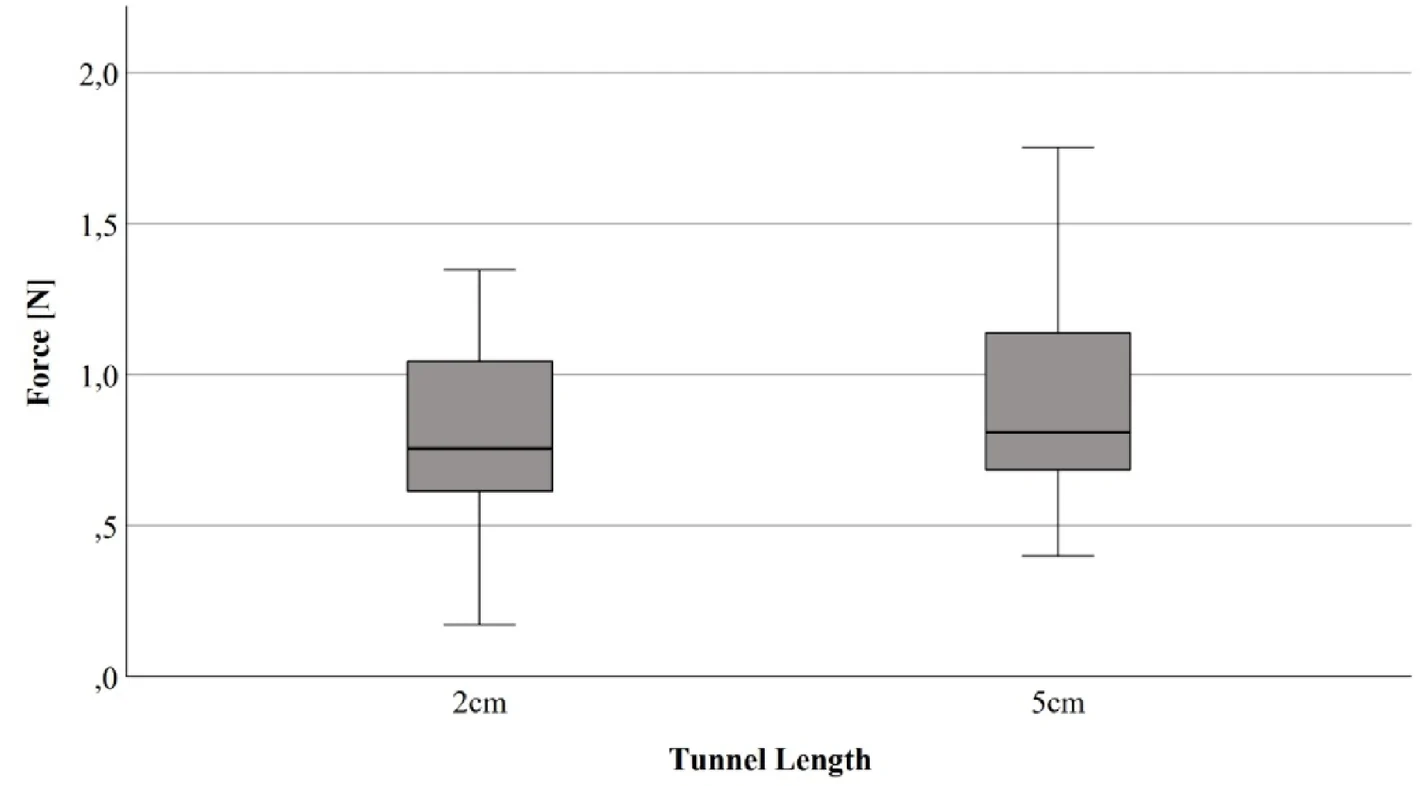
BoxPlot comparing required dislocation forces at 2 cm (*n* = 40) and 5 cm (*n* = 40) tunnel length irrespective of the angle of traction.

The length of the epidural catheter tunnel had no significant effect on the required overall dislocation force when comparing 2 cm versus 5 cm irrespective of the angle of traction applied (*p* = 0.097; Fig. 7).

### Effect of tape type and strip length on required dislocation forces

The efficacy of fixation by a 20 cm adhesive strip was 12 times higher than that of a subcutaneous tunnel. The utilization of 20-cm-long adhesive strips for epidural catheter fixation resulted in significantly higher dislocation forces compared to the use of 10-cm-long adhesive strips (MD = 2.89 N [CI95%: 1.56 to 4.22]; *p* < 0.001). The effect of strip length on the required dislocation force can be estimated by a linear regression model. Thus, an augmentation of 1 cm in strip length results in a concomitant increase in the required dislocation force of 0.29 N [CI95%: 0.09 to 0.49] (Regression equation: Force [N] = 0.513 + 0.289 * Length [cm]; *p* = 0.007; R^2^ = 0.44; Residuals were normally distributed, as heteroskedasticity was identified in the residual analysis (Breusch-Pagan-test *p* = 0.031), HC3 heteroskedasticity-consistent robust standard errors were used).

**Fig. 8:**
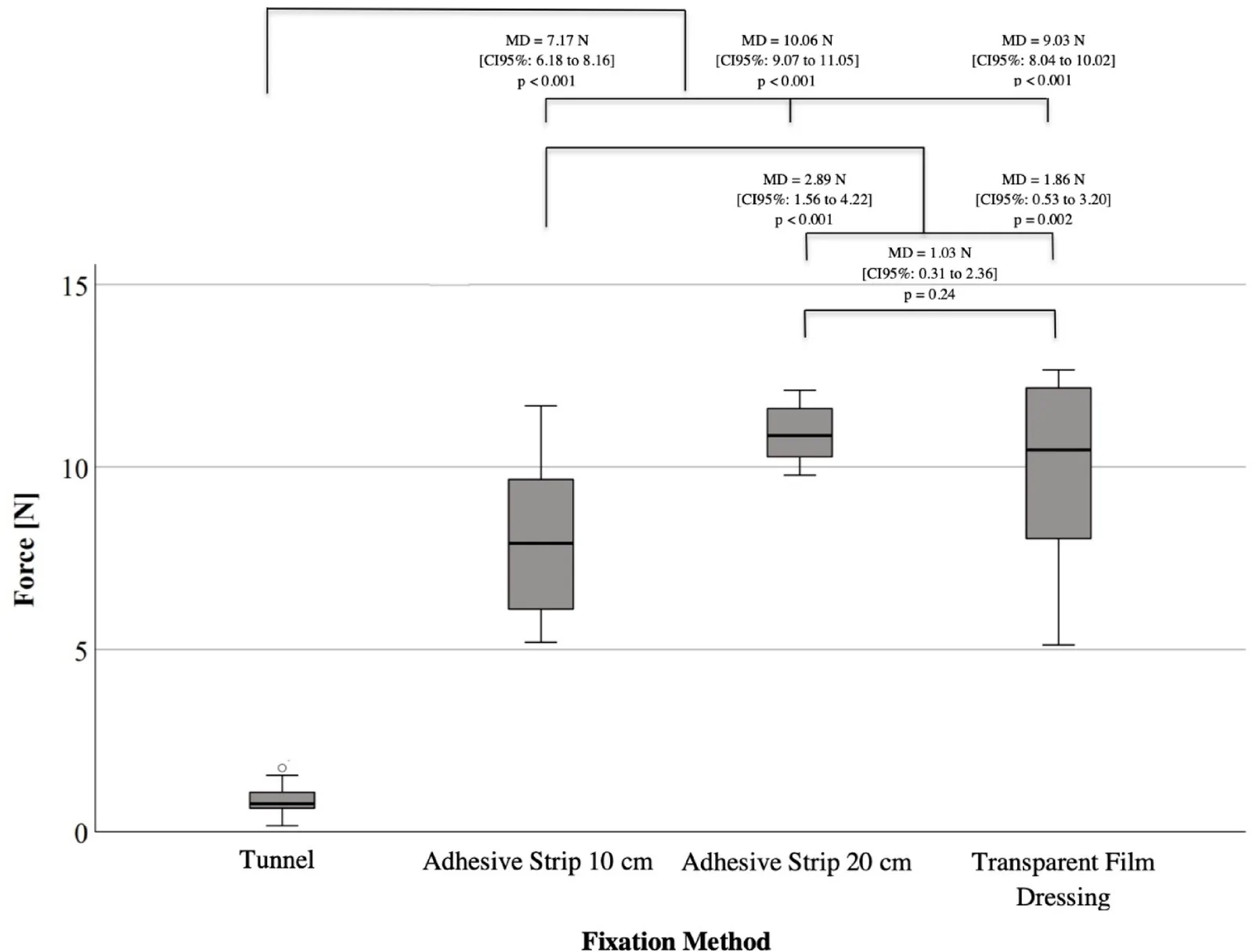
BoxPlot showing required dislocation forces for tunneled epidural catheters in comparison to adhesive strips of 10 cm length (*n* = 8), 20 cm length (*n* = 8) and transparent film dressing (*n* = 8). MD = mean difference.

Fixation with transparent film dressing exhibited considerably higher dislocation forces compared to 10-cm-long adhesive strips (MD = 1.86 N [CI95%: 0.53 to 3.2]; *p* = 0.002) (Fig. 8) and was comparable to the catheter stabilizing effect of a 20 cm adhesive strip (MD = 1.03 N [CI95%: -0.31 to 2.36]; *p* = 0.24).

### Comparison of the dislocation forces with different fixation methods

The mean required force to dislodge a tunneled epidural catheter was 0.86 ± 0.3 N. In comparison, the required forces to migrate the catheter secured by adhesive strips with a length of 10 cm (8.03 ± 2.3 N) and 20 cm (10.92 ± 0.84 N) or by transparent film dressing (9.89 ± 2.65 N) were considerably higher (all p-Bonferroni < 0.001) (Fig. 8).

## Discussion

In clinical practice, epidural catheters are routinely secured using a combination of subcutaneous tunneling and/or adhesive dressings to avoid dislocation and its concomitant adverse effects. Our in vitro study examined the mechanical principles of single components of various catheter fixation methods immediately after application in a porcine model.

The results demonstrate that (i) variations in length and angle of the subcutaneous tunnel demonstrated no impact on the force required for catheter dislocation. (ii) Adhesive fixation, using a transparent film dressing or an adhesive strip applied alongside the catheter, contributed significantly more to catheter stability than subcutaneous tunneling immediately after application and (iii) extending the adhesive strip alongside the catheter resulted in an increase of the force required for dislocation.

High rates of epidural catheter dislocation have been reported in literature, ranging from 36% to 56%^3–8^. The observed variability can be attributed to the heterogeneity in the definitions of the term “dislocation” across the respective studies, as these differ in terms of dislocation length, the distinction between inward and outward dislocation, and the use of imaging methods or clinical functionality tests.

When unintended catheter migration occurs, the criteria for adequate pausing of anticoagulant medication might not be met. The potential consequences of the unintended catheter migration include the occurrence of epidural or spinal hematoma, with a concomitant risk of neurological complications. In cases of severe inward or outward migration, the catheter dislocates from the epidural space, resulting in the loss of analgesia and potentially necessitating the placement of a new epidural catheter^2^. It is evident that each instance of a neuraxial procedure is accompanied by a specific set of inherent risks, which may encompass neurological complications, the potential for infection, or the occurrence of bleeding complications. Therefore, it is imperative to prioritize the optimal catheter fixation method to ensure patient safety and prevent catheter dislocation. Studies examining whether subcutaneous tunneling reduces the risk of dislocation compared to fixation with adhesive dressings alone are not entirely consistent.

In a prospective randomized controlled trial examining 100 tunneled epidural catheters and 113 epidural catheters secured with a standard dressing technique, Burstal et al. demonstrated that subcutaneous tunneling reduces the risk of both inward and outward migration significantly^5^. Interestingly, catheter dislodgement was not related to the duration of catheterization. In contrast, another prospective randomized controlled trial conducted by Abukhudair et al. reported no significant effect of subcutaneous tunneling on the dislocation rate of epidural catheters^10^. Here, the mean number of days for epidural analgesia was slightly lower (2.5 to 2.7 days) compared to Burstal et al. with (3.1 to 3.5 days)^5^. In both studies, the majority of the examined catheters were thoracic epidural catheters. Bougher et al., also compared tunneled catheters to catheters secured with a transparent dressing in a prospective randomized controlled trial. They found no significant effect of tunneling on outward dislocation but observed a significant reduction in inward migration (24% in non-tunneled versus 2.5% in tunneled catheters)^4^. In a subsequent randomized controlled trial Tripathi et al. also observed a non-significant trend towards reduced inward migration when utilizing tunneled catheters, along with a significant reduction in outward dislocation (21% in non-tunneled versus 3% in tunneled catheters)^8^.

To address the heterogeneity of the available evidence and regarding the complexity of catheter stability in clinical practice, influenced by multiple factors, we conducted in this first approach a basic mechanical assessment of different characteristics of the subcutaneous tunnel and adhesive dressing techniques using an experimental model. Here, subcutaneous tunneling of epidural catheters resulted in an average required force of 0.86 N to induce dislocation. This finding is consistent with the results reported by Byrne et al., who conducted a study on the dislocation of peripheral nerve catheters in a porcine model. Their study indicated that a mean dislocation force of 1.16 N was necessary for tunneled catheters^12^. Furthermore, we could demonstrate that neither the length nor the angle of the tunnel exhibited a substantial impact on the required dislocation force (apart from the marginally significant difference observed between 135° and 0° at a 5 cm tunnel length (*p* = 0.049) assuming an expected clinically relevant difference of 10%. This finding contradicts our original hypothesis, which was based on fundamental physical principles of friction. According to these principles, a longer tunnel or a steeper tunnel angle would increase the force required for dislocation by enhancing friction. To the best of our knowledge, no explanation for this result could be found in the literature. We assume that the subcutaneous adipose tissue in which the catheter is located within the tunnel exhibits only minimal friction and, at least immediately after placement, functions as a lubricant for the catheter. Therefore, the length or angle of the tunnel has only a limited effect on the immediate fixation process. However, the possibility that smaller differences between individual tunnel angles may have remained undetected due to limited statistical power cannot be excluded.

In the evaluation of the adhesive-based fixation methods, catheter fixation with a transparent film dressing or an adhesive strip of 10–20 cm in length applied alongside the catheter significantly enhanced immediate catheter stability in our study. However, comparing our required pull-out forces to the results reported by Hakim et al., securing epidural catheters with various film dressings to synthetic skin^13^, we detected a slightly reduced effect of adhesive fixation methods on catheter stability. This reduced adhesiveness could be attributed to the presence of moisture in the porcine skins, in contrast to the synthetic skin utilized by Hakim et al. These differences observed already in model-based experiments underscore the complexity of catheter stabilization achieved through adhesive techniques in clinical practice. Furthermore, the efficacy of fixation methods depends significantly on the length of the adhesive strip, improving with an increase in strip length. These differences in length of the fixation method might be an additional reason justifying the slight differences in dislocation forces between the two studies.

Our study demonstrated that standardized physical principles do not necessarily translate into the expected outcomes even in a biological ex-vivo model, due to the influence of multiple interacting factors. Consequently, extrapolating findings from a biological model to clinical practice is even more challenging. Here, besides the length and angle of the tunnel, “long-term” effects of vital subcutaneous tissue around the epidural catheter might play an even more important role in catheter stability. Wound healing might increase catheter stability, whereas leaking fluids, moisture, or wound fluid might further affect adhesion. Furthermore, skin elasticity and complex forms of traction forces as repetitive small traction events, twisting, bending or multidirectional loading might affect the different tunnel properties with their effect of fixation in varying ways. Additionally, there are a multitude of factors, influencing the adhesiveness of strips. Skin elasticity, perfusion, sweating, and skin moisture may have a significant impact on catheter stability, particularly regarding the duration of use, where time may alter adhesiveness. Furthermore, mechanical stress could reduce the contact surface of adhesive strips and the adhesive’s performance can be affected by various physical factors, such as temperature, humidity, or light exposure.

Additionally, further specialized devices like LockIt Plus^14^, StatLock, and Epi-Fix^15^ or other techniques as use of the Humble ECG electrode or fixation with a strip of adhesive foam secured with a suture^16^ were not investigated yet.

Therefore, any extrapolation of our findings to the clinical context should be undertaken with utmost caution, particularly as only the short-term behavior of the fixation methods under simple mechanical loading was assessed. However, this first systematic approach provides initial insights into the short-term effects of different fixation methods and, in particular, into the impact of variations in subcutaneous tunnel parameters.

Addressing limitations of the study, first, this was a bench study in a porcine model. Tissue characteristics may vary between species and undergo changes postmortem. This approach cannot adequately account for physiological skin-related processes that may influence catheter stability over time, namely healing, production of wound fluids or sweating. Furthermore, in-vivo factors such as skin moisture, temperature, and varying mechanical stress may influence the adhesiveness of the strips. However, conducting preliminary trials in patients to assess the efficacy and safety of different angles and lengths of subcutaneous tunnels would be unethical due to the potential for significant patient discomfort. Given the necessity to standardize both the precise angle and length of the tunnel, as well as to circumvent potential confounders arising from interindividual differences such as skin thickness, the porcine model was deemed a more suitable subject for examining the fundamental physical principles. However, the absolute forces required for dislocation must be transferred to clinical practice with caution as well as the long-term effects of the different fixation methods. Furthermore, the potential for synergistic effects between the single fixation components is a possibility; however, this was not the objective of the present study. Our study was conducted with the primary aim of understanding the contribution of single components.

Moreover, blinding the observers was not possible in our experimental setup. However, at least the recorded force data was analyzed by a blinded evaluator to reduce possible biases.

To conclude, we demonstrated that length or angle plays a minor role in overall fixation effect of the subcutaneous tunnel during immediate pull-out forces. The “short-term” effect of adhesive dressings compared to subcutaneous tunneling on catheter stability was higher. Clinical applicability of our results remains difficult. However, this study is a first step evaluating the different fixation methods and its various applications in a systematic way. Further in-vivo studies are necessary to characterize the role of subcutaneous tunneling in catheter stability compared to other fixation techniques, especially according to the long-term effects of catheter stabilization. These studies should incorporate clinical endpoints, such as clinically relevant catheter dislocation (> 2–3 cm), insufficient analgesia, and patient comfort regarding the different procedures, as well as infectious and bleeding complications.

## Supplementary Information

Below is the link to the electronic supplementary material.

Supplementary Material 1

## Data Availability

All datasets generated in this study are available from the corresponding author on reasonable request. The data is not publicly available.
